# Using variable importance measures to identify a small set of SNPs to predict heading date in perennial ryegrass

**DOI:** 10.1038/s41598-017-03232-8

**Published:** 2017-06-15

**Authors:** Stephen L. Byrne, Patrick Conaghan, Susanne Barth, Sai Krishna Arojju, Michael Casler, Thibauld Michel, Janaki Velmurugan, Dan Milbourne

**Affiliations:** 1 Teagasc, Crop Science Department, Oak Park, Carlow Ireland; 2 Teagasc, Animal and Grassland Research and Innovation Centre, Oak Park, Carlow, Ireland; 3 Department of Botany, Trinity College Dublin, Dublin 2, Dublin, Ireland; 4 Department of Agronomy, University of Wisconsin-Madison, Madison, WI 53706 USA; 5 USDA-ARS, U.S. Dairy Forage Research Center, Madison, WI 53706-1108 USA

## Abstract

Prior knowledge on heading date enables the selection of parents of synthetic cultivars that are well matched with respect to time of heading, which is essential to ensure plants put together will cross pollinate. Heading date of individual plants can be determined via direct phenotyping, which has a time and labour cost. It can also be inferred from family means, although the spread in days to heading within families demands roguing in first generation synthetics. Another option is to predict heading date from molecular markers. In this study we used a large training population consisting of individual plants to develop equations to predict heading date from marker genotypes. Using permutation-based variable selection measures we reduced the marker set from 217,563 to 50 without impacting the predictive ability. Opportunities exist to develop a cheap assay to sequence a small number of regions in linkage disequilibrium with heading date QTL in thousands of samples. Simultaneous use of these markers in non-linkage based marker-assisted selection approaches, such as paternity testing, should enhance the utility of such an approach.

## Introduction

Perennial ryegrass (*Lolium perenne*) is the primary forage used in many temperate agriculture regions, and in some countries completely underpins the dairy and livestock sectors. Commercial varieties are developed by intercrossing selected genotypes in isolation. Genetic gain in perennial ryegrass is generally low in comparison to grain crops, and in recent years there has been a focus on using genomic selection to help accelerate genetic gain. A few studies have reported on the accuracy of using genomic information to predict a range of phenotypes in perennial ryegrass, including heading date^1–3^.

Heading date indicates the onset of anthesis, and results in a reduction in forage quality due to a higher stem to leaf ratio. During official testing, candidate varieties are typically classified and evaluated under different heading groups. Heading date is also used as a trait to assess distinctiveness, uniformity, and stability (DUS). Predictive accuracies for heading date of between 0.84 and 0.90 have been achieved using genomic data^1^. There has been mixed success in using Genome Wide Association Analysis (GWAS) to identify Quantitative Trait Loci (QTL) for heading date in perennial ryegrass, with one study failing to identify any significant QTL^4^ and another identifying a limited number of QTL accounting for just 20.3 percent of the phenotypic variance^1^. There are many reasons for this, including insufficient marker density given the rapid decay of LD, very rare alleles, and the correlation of heading date with population structure. A number of bi-parental mapping populations have been used in classical QTL studies and identified a number of moderate affect QTL on different linkage groups^5–15^. However, there is nothing in the literature describing the conversion of markers linked to these QTL into molecular assays for the prediction of heading date in a broader set of material.

Heading date is visually assessed and therefore relatively straight forward to evaluate, has a high heritability and is generally used as a model trait. However, it is also a trait of crucial importance in variety development. Perennial ryegrass varieties are sold as synthetic cultivars, and when selecting individual genotypes for synthetics it is vital that they are matched with respect to heading date or they will not cross pollinate. Furthermore, if the range in heading date within a variety is too large they will fail DUS. Heading date can be determined directly on individuals in spaced plant nurseries. Accurate prediction of heading date with molecular markers would enable selection of plants that are matched with respect to heading date from within high performing families without any prior phenotypic evaluation of single plants. Even within families there is significant variation for heading date, and family means are not always accurate in predicting heading date of individual plants.

We have evaluated heading date in a large population of single plants and used genoyping-by-sequencing to evaluate genotypes. Genotypes were used to predict heading date and variable selection strategies enabled the identification of marker subsets with high predictive power. These marker subsets are suitable for the development of cost effective molecular assays to predict heading date.

## Results

### Heading date variance within training population

**Figure 1:**
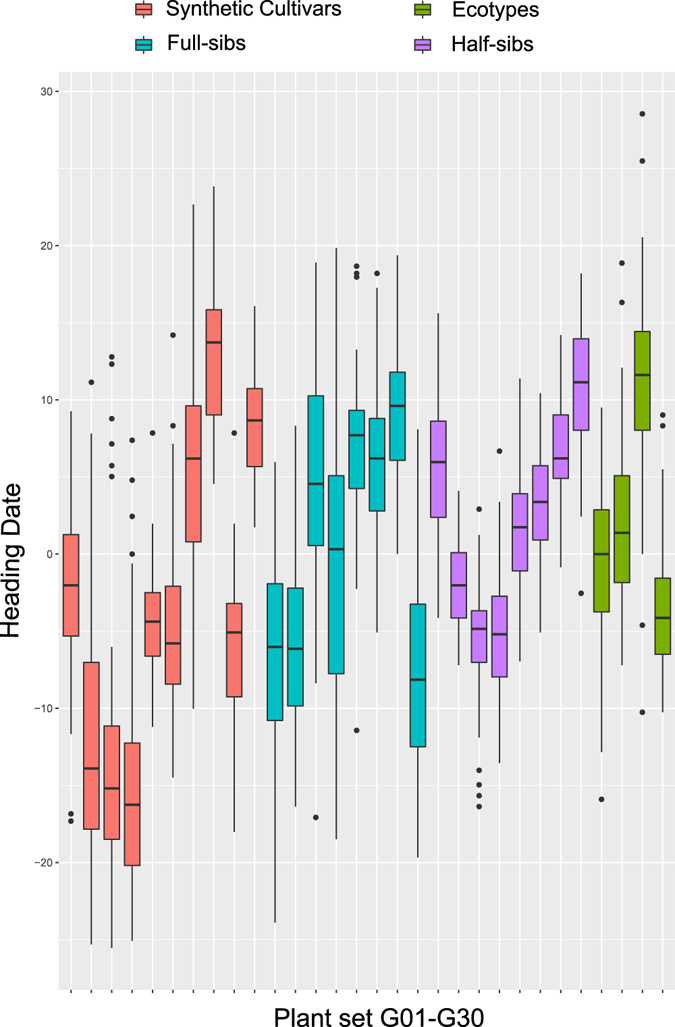
Heading date scores across populations. Boxplots show the conditional modes calculated for each individual and grouped by family, cultivar or ecotype.

The complete training population consists of plants taken from synthetic cultivars, full- and half-sib families, and ecotypes. These were scored for heading date across two replicates and over two years, and conditional modes for heading date were calculated (Fig. 1). The greatest range in heading dates was observed within the synthetic cultivars. As can be seen from Fig. 1, there is substantial within family/cultivar variation for heading date. The broad sense heritability (repeatability) was calculated as 0.91.

### Training population genotypes

**Figure 2:**
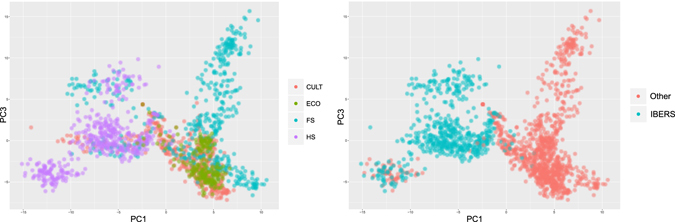
Principle Component Analysis of complete population based on an individual plants genotype. Individual plants are colored according to mating type on the left. On the right, individual plants are colored according to whether or not they originate from an IBERS bred cultivar.

Overall, 1582 plants were genotyped using a genotyping-by-sequencing strategy and we identified 217,563 SNPs with a minor allele frequency of at least 0.01. Unsurprisingly, the genomic relationship matrix generated with the SNP data shows strong relationships between individual plants from the same family, cultivar, or ecotype (Supplementary Fig. S1). The first principle component in a Principle Component Analysis (PCA) accounted for 10.4 percent of the variation (Supplementary Fig. S2), and the cumulative variation accounted for by the first three principle components was 15.8 percent. We see little distinction between ecotypes and cultivars, with the clearest separation occurring between plants directly originating from IBERS bred varieties (IBERS, Aberystywth University, UK) or from families with IBERS parentage (Fig. 2). One half-sib family with unclear parentage did cluster with the IBERS varieties, but it is likely to have originated from IBERS bred material. The strong relationship among IBERS plant material is also evident in the genomic relationship matrix (Supplementary Fig. S3).

The complete population was sub-divided into four smaller populations, (i) cultivars, (ii) half-sibs, (iii) full-sibs, and (iv) ecotypes, to enable a comparison of predictive ability across different training population designs. To ensure an appropriate marker set for each sub-population we re-analysed the sequence data and identified an SNP set for each population (Table 1). In all populations linkage disequilibrium decayed towards background levels over very short distances (Supplementary Fig. S4). This is consistent with previous reports of LD in various perennial ryegrass populations^1, 2, 16^.

**Table 1:** Composition of the training population and SNP numbers identified within each sub-group.

Population	No. Individuals	No. SNPs (MAF 1%)	No. SNPs (MAF 5%)
Complete	1582	217563	138644
Synthetic cultivars	445	135674	81658
Half-sib families	448	262472	191519
Full-sib families	479	232864	153295
Ecotypes	210	263392	177222

A new round of SNP calling was performed for each sub-group.

### Predictive ability for heading date

**Figure 3:**
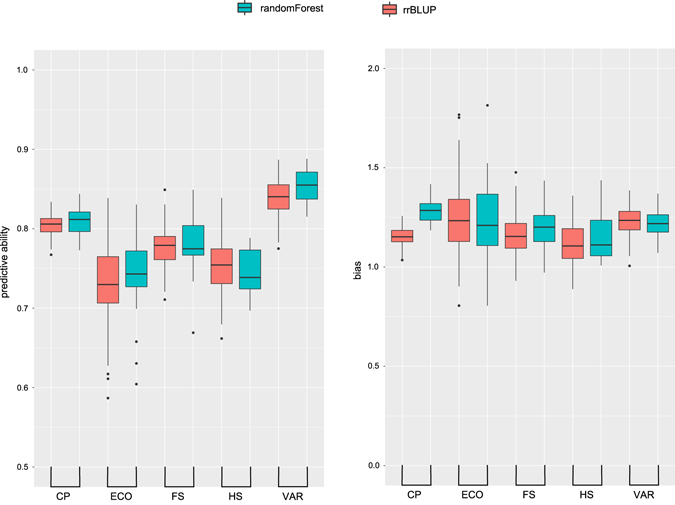
Predictive ability for heading date. Predictive ability (on the left) is measured as the correlation between the conditional modes for heading date and the predicted values. The bias (on the right) is *β* from a regression of predicted phenotypes (*x*) vs observed phenotypes (*y*).

Overall, predictive ability for heading date was quite high with median predictive abilities ranging from 0.73 to 0.86 (Fig. 3), corresponding to predictive accuracies ranging from 0.76 to 0.90. Using the complete population as a training set, the median predictive ability was 0.81 with both statistical approaches (rrBLUP and random forest regression), although the bias was higher with random forest (Fig. 3). The highest predictive abilities were achieved when training and predicting within synthetic cultivars, with a slightly higher predictive ability using random forest (0.86) over rrBLUP (0.84). The higher predictive ability within synthetic varieties is likely related to greater variation for heading date, and in particular the presence of many early flowering phenotypes (Fig. 1).

**Figure 4:**
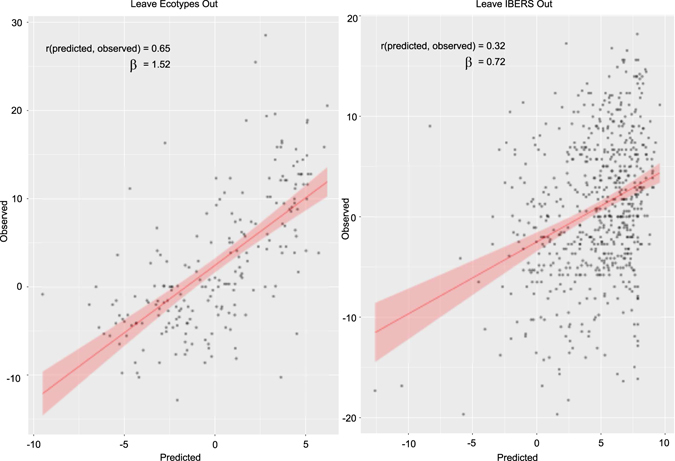
Predictive ability when predicting from unrelated material using the complete SNP set. Scatter plots of predicted vs. observed phenotype when predicting IBERS plant phenotypes with models trained on non-IBERS plants (right), and when predicting ecotype phenotypes with models trained on non-ecotype plants (left).

We also evaluated predictive ability when leaving related material out. In the first evaluation we performed training within the breeding material and predicted within the ecotypes (Fig. 4), resulting in a drop in predictive ability to 0.65. The clearest differentiation within the complete population is among IBERS derived plants and other plants (Fig. 2). When predicting IBERS material from other material there was a large reduction in predictive ability to 0.32. This is not dissimilar to the drop in predictive ability observed when predicting across breeds in animal genomic selection.

### Variable importance measures

**Figure 5:**
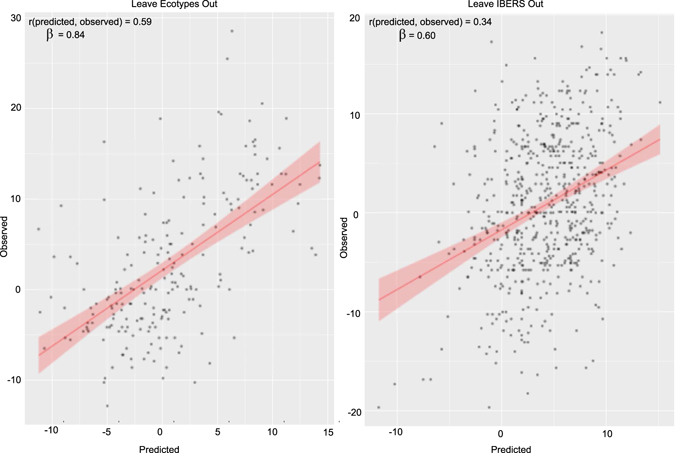
Predictive ability for heading date using selected vs random variables. Selected variables were identified on a training set using permutation-based variable selection measures, predictive were models developed with these variables and used to predict phenotypes in the test set (results of 100 iterations of Monte Carlo cross-validation are presented). Predictive ability (on the left) is measured as the correlation between the conditional modes for heading date and the predicted values. The bias (on the right) is *β* from a regression of predicted phenotypes (*x*) vs observed phenotypes (*y*).

We used permutation-based variable selection measures to rank and select a sub-set of variables (SNPs) for prediction (Supplementary Data S1). Variables were ranked according to the mean decrease in accuracy and we selected the top 50 for predictive modeling. The predictive ability using 50 variables was similar to the predictive ability using the complete set. In contrast, the predictive ability with 50 random variables was substantially lower and with higher bias (Fig. 5). Despite this there was still some predictive power (median predictive ability of 0.42) when using 50 random variables, indicating that small SNP sets are able to capture some of the population structure correlated with heading date. The adjusted coefficient of multiple determination in a linear regression using the 50 selected variables was 0.58, indicating the 50 selected SNPs can explain much of the variability in heading date in this population. We used cross validation with 70:30 split between training and test data and identified the top 50 SNPs at each iteration for use in a linear regression to predict heading date in the test set. The median predictive ability was 0.74 and the median *β* was 0.96. The Root Mean Square Error (RMSE) (3) was calculated at each iteration and the median was 6.0. The range in heading dates (conditional modes) was 51.03, and RMSE corresponding to 6.0 days may be an acceptable prediction error when selecting plants to combine for a poly-cross.

**Figure 6:**
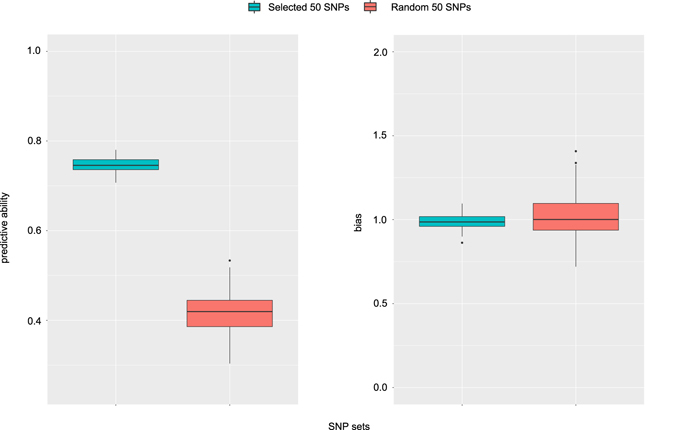
Predictive ability when predicting from unrelated material using the selected SNP set. Scatter plots of predicted vs. observed phenotype when predicting IBERS plant phenotypes with variables selected and models trained on non-IBERS plants (right), and when predicting ecotype phenotypes with variables selected and models trained on non-ecotype plants (left).

We already observed a significant drop in predictive ability to 0.32 when predicting in IBERS material from other material. However, using only the 50 selected SNPs the predictive ability slightly improved to 0.34 (Fig. 6), although the predicitve ability was slightly lower when predicting in ecotypes from other material.

### Genetic architecture of heading date

We used the perennial ryegrass draft genome^17^ to extract the genomic scaffolds containing the 50 top ranked SNPs after variable importance measures were calculated using the complete population. Genes located within these scaffolds were characterized (Supplementary Data S2). The 50 SNPs were located within 39 scaffolds that had been annotated with 56 genes. We were able to determine position on a genetic linkage map for 17 of the scaffolds using the GenomeZipper^17, 18^. Of the scaffolds that could be anchored, there was some clustering (3 or more) at positions on Linkage Groups (LG); 2 (79.7–81.9 cM), 3 (36.4–43.3 cM), 4 (60.7–64.0), and 7 (44.7–48.4 cM). The scaffolds on LG2 and LG7 cluster in regions with key genes involved in the timing of flowering in other species. This includes TFL1 on LG2 (79.8 cM) and FT and CO on LG7 (43–44 cM)^4^. TFL1 is a repressor of flowering that is down regulated in perennial ryegrass following a period of vernalisation. In contrast FT and CO are both promotors of flowering with CO acting upstream of the key floral activator FT. One scaffold had a perennial ryegrass protein that was previously shown to be an orthologue of PRR37 from rice^4^ and was anchored to LG2 at 12.4 cM. One of the other 39 scaffolds was also anchored to this region. PRR37 is a Pseudo-Response Regulator that was found to underlie a major heading date QTL in rice, and it was shown that natural variation in PRR37 likely contributed to the expansion of rice cultivation to temperate regions^19^. Alone, SNPs in the two scaffolds within this region can explain 18 percent of the phenotypic variation for heading date.

## Discussion

Accurate heading date information on individual plants is vital to forage breeders to ensure selected plants cross pollinate. It enables selection of synthetic components with comparable heading dates, therefore ensuring sufficient cross-pollination and seed yield. Here, we used a large panel of genome wide SNPs to predict heading date with an accuracy of up to 0.86, in agreement with a previous study using F2 families^1^. Currently, heading date of individual plants is evaluated directly in spaced plant nurseries, requiring an additional year of spaced plant evaluations. Inferring heading date from family means is difficult as there is substantial within family variation for heading date (Fig. 1). Therefore, a low cost marker system to predict heading date would be beneficial.

Using variable importance measures we have been able to identify a list of 50 SNPs that have predictive power comparable to the complete SNP set (217,563). This is a two step process akin to marker assisted selection, in stage one we are identifying the SNPs with a large effect on predictive ability, and in stage two we are using these for prediction. In many cases the SNPs we identified as important for prediction were proximal to orthologues of proteins with key roles in the timing of flowering in other species. Variable selection strategies can be used as an approach to reduce the genotyping cost and are expected to outperform approaches that evenly distribute effects across the entire genome in cases where heritability is high, number of causal mutations is small relative to the sample size, and where LD only extends to very short distances^20^. All three of these assumptions are expected to be met when predicting heading date in perennial ryegrass. A recent study of flowering time and spike grain number in wheat indicated that genomic prediction methods effectively capturing LD between markers and traits outperformed other models when training and testing material were unrelated^21^.

It is now possible to design cheap molecular assays focused on amplification and sequencing of a few hundred target regions (up to 500). The cost per sample is greatly reduced using dual barcoding systems that enable the multiplexing of 1000 s of samples^22^. Genotyping at 192 loci in 2068 samples was achieved at a cost of $3.98 per sample including DNA isolation and sequencing. The selected SNPs identified above can be developed into such an assay, and complemented with SNPs predictive for other traits. It is feasible that a similar strategy of selecting SNPs based on variable importance measures will work for traits such as crown rust resistance, and quality. In addition to linkage based applications, there is also the potential to use these markers in non-linkage based marker assisted selection strategies. The first example of such an approach involves using markers for paternity testing in half-sib recurrent selection schemes. In this case molecular markers are used to determine the paternal parent when selecting within the top performing half-sib families, which increases the selection gains and removes the burden of maintaining maternal parents through evaluations. The value of such an approach has already been demonstrated for red clover^23^, and should also be relevant when selecting for forage yield in perennial ryegrass half-sib recurrent selection schemes. In such a scheme the markers would be used to predict or generate breeding values for traits such as heading date and crown rust resistance, while also identifying the paternal parent enabling increased selection gains for forage yield. The ideal requirements for assigning paternity is a marker system that has independent, highly allelic co-dominant markers with many low frequency alleles^24^. The sequenced amplicons can easily be converted into such a multi-allelic marker system to assign paternity, especially considering all the potential pollen donors are known and can be genotyped.

Another potential non-linkage based application of these molecular markers is to maximize diversity when selecting synthetic components from top performing families. Full-sib recurrent selection schemes involve evaluating F2 families for forage yield over a number of years followed by selection of individual plants from within top-performing families to make synthetics. As discussed above, genotyping individuals within these families would enable accurate prediction of heading date and potentially generate breeding values for traits such as crown rust resistance and forage quality. On top of this, the markers could also be used to maximize diversity among parents used in the synthetic polycross through selection of the most genetically diverse individuals from top performing families. A study conducted using AFLP markers in perennial ryegrass has already demonstrated that using markers to increase diversity among polycross parents can lead to increased dry matter yields^25^. As discussed above, the sequenced amplicons can easily be converted to a multi-allelic marker system.

We have identified a relatively small number of SNPs with excellent predictive ability for heading date. We envisage being able to combine these with similarly small sets of SNPs that can predict crown rust and quality, enabling the development of a cheap molecular assay that can be applied in breeding schemes. This can be applied in populations derived from the training material described here. Furthermore, using the markers in non-linkage based approaches such as paternity testing and to maximise diversity among polycross parents will enhance the benefits of such an assay in both half-sib and full-sib recurrent selection schemes.

## Methods

### Populations, field trials and phenotypic analysis

The training population consists of up to 60 plants from each of ten synthetic cultivars, eight full-sib families, eight half-sib families, and four ecotypes (Table 2). Plants were evaluated as spaced plants in a partially balanced incomplete block design with two replicates at Oak Park, Carlow, Ireland. Each replicate was divided into 30 blocks each consisting of 60 test genotypes (2 test genotypes from each of the 30 families) and 5 check genotypes (coming from the varieties Donard, Premium, Spelga, Gilford, and Portstewart). The five check genotypes were clonally propagated and are identical across all blocks. Altogether each of the two replicates had 1,950 plants that were subjected to infrequent cutting (four cuts per year), and heading date was evaluated over two years (2014 and 2015). Heading date was scored from April 1st until the first spike had emerged from three tillers of an individual spaced plant. Variance components for heading date were estimated using the R package lme4^26^. The variance components were used to calculate the broad-sense heritability, estimated as:

1 $${h}_{B}^{2}=\frac{{\sigma }_{g}^{2}}{({\sigma }_{g}^{2})+({\sigma }_{gy}^{2})\mathrm{/2}+({\sigma }_{res}^{2})\mathrm{/4}}$$

where ${\sigma }_{g}^{2}$, ${\sigma }_{gy}^{2}$, and ${\sigma }_{res}^{2}$ are estimates of variance components for genotypes, genotype by year interaction, and residuals respectively. Conditional modes (also referred to as best linear unbiased predictors of the random effects) were estimated for each genotype in lme4 using genotype, and blocks within replicates as random effects, and year and checks as fixed effects. Conditional modes were returned using the ranef extractor in lme4. These were used to develop models to predict heading date from genomic information.

**Table 2:** Pedigree of the plant material that makes up the training population.

Ref. ID		
**Cultivars**	**Name**	
G01	Aberstar*	
G02	Arrow	
G03	Commando	
G04	Genesis	
G05	Impact	
G06	ONE50	
G07	Tyrella	
G08	Malambo	
G09	Boyne	
G10	Glenroyal	
**Full-sib families**	**Parent 1**	**Parent 2**
G11	Pastour	Genesis
G12	Solomon	Tyrella
G13	Jumbo X Tyrone cross	Portsewart X Fennema cross
G14	(Donard X Morgana) X (Donard X Corbiere) cross	Portsewart X Fennema cross
G15	Profit X Hercules cross	Jumbo X Tyrone cross
G16	AberAvon*	Twystar
G17	Tyrconnell	Majestic
G18	AberSilo*	Shandon
**Half-sib families**	**Maternal parent**	**Paternal parent**
G19	Jumbo	Aberdart*
G20	Dorset	Aberdart*
G21	Spelga	PNI
G22	Premium	Aberzest*
G23	Stratos	Aberzest*
G24	Lasso	Aberzest*
G25	Cornwell	Aberzest*
G26	Romark	Aberchoice*
**Ecotypes**	**Genebank ID**	**County/Country**
G27	IRL-OP-02007	Cork/Ireland
G28	IRL-OP-02018	Wicklow/Ireland
G29	IRL-OP-02491	Wexford/Ireland
G30	IRL-OP-02572	Kildare/Ireland

*IBERS bred varieties (IBERS, Aberystywth University, UK).

### Genotyping

We used a genotyping-by-sequencing approach that followed the protocol developed by Elshire *et al*.^27^. Briefly, genomic DNA was isolated from each individual, digested with ApeKI, samples were grouped into libraries, amplified, and sequenced on an Illumina HiSeq 2000. After sequencing, adaptor contamination was removed with Scythe^28^ with a prior contamination rate set to 0.40. Sickle^29^ was used to trim reads when the average quality score in a sliding window (of 20 bp) fell below a phred score of 20, and reads shorter than 40 bp were discarded. The reads were demultiplexed using sabre^30^ and data from each sample was aligned to the perennial ryegrass reference genome^17^ using BWA^31^. The Genome Analysis Tool Kit (GATK)^32^ was used to identify putative variants in the complete population of 1582 plants. The plants were then divided into four smaller populations (i) full-sib families, (ii) half-sib families, (iii) ecotypes, and (iv) synthetic cultivars, and variants were identified in each of these. Only genotype calls with a phred score of 30 (GQ, Genotype Quality), and only variant sites with a mean mapping quality of 30 were retained. In all cases we used a minimum minor allele frequency threshold of 1% when identifying SNPs, and any SNPs with greater than 50% missing data points were eliminated.

### Evaluating genetic structure of population

Principle Component Analysis (PCA) was carried out in R^33^ using a reduced SNP set with less than 25% of missing data points and a Minor Allele Frequency (MAF) of at least 1%. This left 6,469 SNPs, and missing data was imputed using mean imputation (MI). The additive genomic relationship matrix was generated using the complete SNP set (217,563) with the A.mat function of the R package rrBLUP^34^, and missing values were imputed with MI.

Linkage disequilibrium (LD) was assessed in the complete population and the four sub-populations. We identified SNPs located within a single genomic scaffold, and calculated the inter SNP distance and the squared correlation of the allele counts in Plink 1.9^35^, based on the maximum likelihood solution to the cubic equation^36^.

### Genomic prediction models

We tested two statistical models for genomic prediction, Ridge Regression BLUP and the machine learning algorithm Random Forest Regression. Ridge Regression BLUP was performed with the R package rrBLUP^34^. rrBLUP was used to solve mixed models of the form

2 $$y=\mu +Xg+\varepsilon $$

where y is the vector of conditional modes for heading date, *μ* is the overall mean, X is the marker matrix, g is a matrix of marker effects, and *ε* is a vector of residual effects. Random Forest Regression was performed with the R package’randomForest’^37^, with the following settings: number of variables (p) at each split = p/3, number of trees = 500, and minimum node size = 5. Random forest regression generates decision trees from subsets of individuals selected by bootstrapping. For each bootstrap sample a regression tree is grown and at each split in the tree a subset of variables (e.g. p/3) is selected at random and used to identify the best split. This is repeated for each bootstrap sample and the trees are averaged. We also used the randomForest package to generate variable importance measures. Permutation based measures of variable importance were calculated on the training set and ranked according to the mean decrease in accuracy. The settings used in randomForest are identical to those described above. The top 50 variables were selected and used for model development with rrBLUP and prediction in the test set. We also performed variable importance measures on the complete data set and identified the variables for anchoring on the perennial ryegrass draft genome (reported in the section “Genetic architecture of heading date”).

We evaluated the accuracy of genomic based prediction in the various subsets of the population (complete population, half-sibs, full-sibs, ecotypes, and synthetic cultivars). The purpose was to compare the effect of training population design on the accuracy of genomic prediction. We performed Monte-Carlo cross-validation by dividing the populations into training and testing sets by randomly assigning (without replacement) 70% of plants to the training set and the remainder to the testing set. In the case of rrBLUP we performed 100 iterations, and in the case of randomForest we performed 25 iterations. Predictive ability was calculated as the Pearsons correlation between the observed and predicted phenotypes. We also performed multiple linear regression by dividing the samples into training and testing sets (70:30) and using the training set to identify and rank variables with the randomForest package as described above. The regression models were built using the top 50 ranked SNPs, and we performed 65 iterations of Monte-Carlo cross validation. The predictive ability, and Root Mean Sqaure Error (RMSE) were calculated at each iteration. The RMSE was calculated as

3 $$RMSE=\sqrt{\frac{1}{n}\sum _{i=1}^{n}{({y}_{i}-{\hat{y}}_{i})}^{2}}$$

where *y*_*i*_ is observed values and ${\hat{y}}_{i}$ are predicted values and the difference between them is the prediction error. Predictive accuracy was estimated by dividing the predictive ability by the square root of the heritability (1). In addition to cross-validation via random assignment of plants into training and testing wet, we also performed cross-validation by leaving specific groups of plants out of the training set. In the first of these cross-validations we left the four ecotypes (209 plants) out of the training set and used them for testing. In the second of these cross-validations we left all material originating from IBERS (628 plants) out of the training set and used them for testing.

### Data Availability

Phenotype data for heading date measured on the population is available on Figshare (https://doi.org/10.6084/m9.figshare.4814740.v1).

## Electronic supplementary material

Supplemental Information

Dataset 1

Dataset 2
