# Supplementary material for: Using variable importance measures to identify a small set of SNPs to predict heading date in perennial ryegrass
Source: Sci Rep. 2017 Jun 15;7:3566. doi: 10.1038/s41598-017-03232-8 (PMC5472636; doi:10.1038/s41598-017-03232-8)
Supplement: Supplementary file 1 — Supplemental Information [file 41598_2017_3232_MOESM1_ESM.pdf]

Using variable importance measures to identify a small set of SNPs to predict heading date in perennial ryegrass.

Stephen L. Byrne[1,*], Patrick Conaghan[2], Susanne Barth[1], Sai Krishna Arojju[1,3], Michael Casler[4, 5], Thibauld Michel[1], Janaki Velmurugan[1], and Dan Milbourne[1]

[1]Teagasc, Crop Science Department, Oak Park, Carlow, Ireland
[2]Teagasc, Animal and Grassland Research and Innovation Centre, Oak Park, Carlow, Ireland
[3]Department of Botany, Trinity College Dublin, Dublin 2, Dublin, Ireland
[4]Department of Agronomy, University of Wisconsin-Madison, WI 53706, USA
[5]USDA-ARS, U.S. Dairy Forage Research Center, Madison, WI 53706-1108 USA

[*]stephen.byrne@teagasc.ie

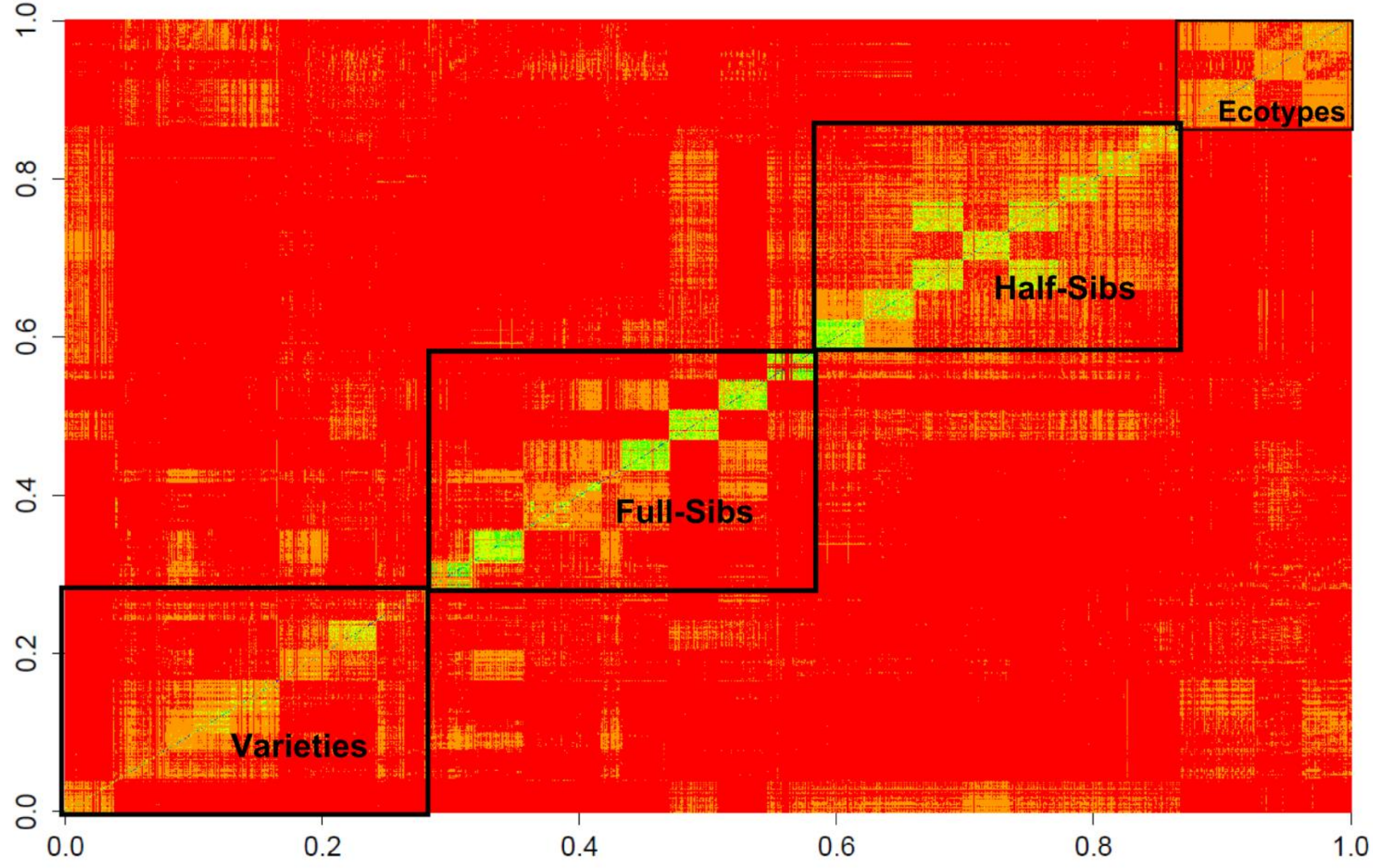

Supplementary Figure 1: Genomic relationship matrix. Genomic relationship matrix generated with SNP data, showing strong relationships between individual plants from the same family (individual plants are ordered according to the family they belong to, G01-G30).

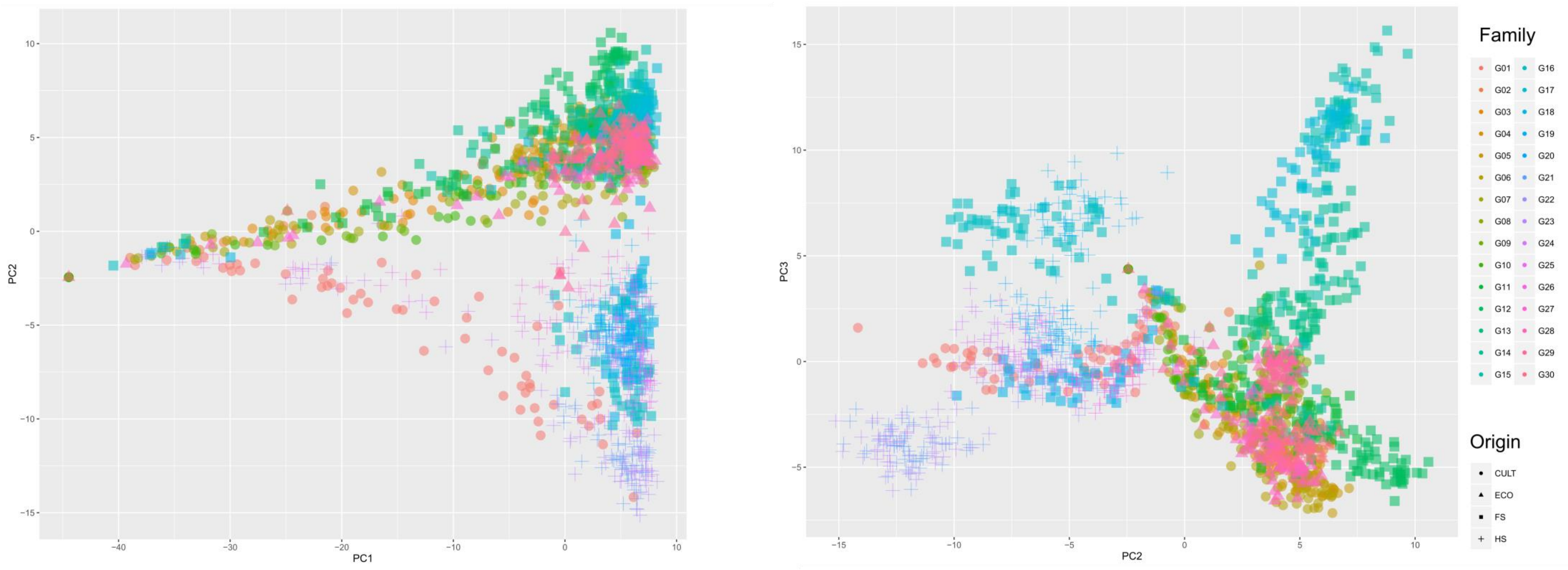

Supplementary Figure 2: Principle Component Analysis (PCA) with SNP data. PC1 vs. PC2 is shown on the left and PC2 vs. PC3 is shown on the right. The first PC accounted for 10.4 percent of the variation and the first three PC accounted for 15.8 percent of the variation.

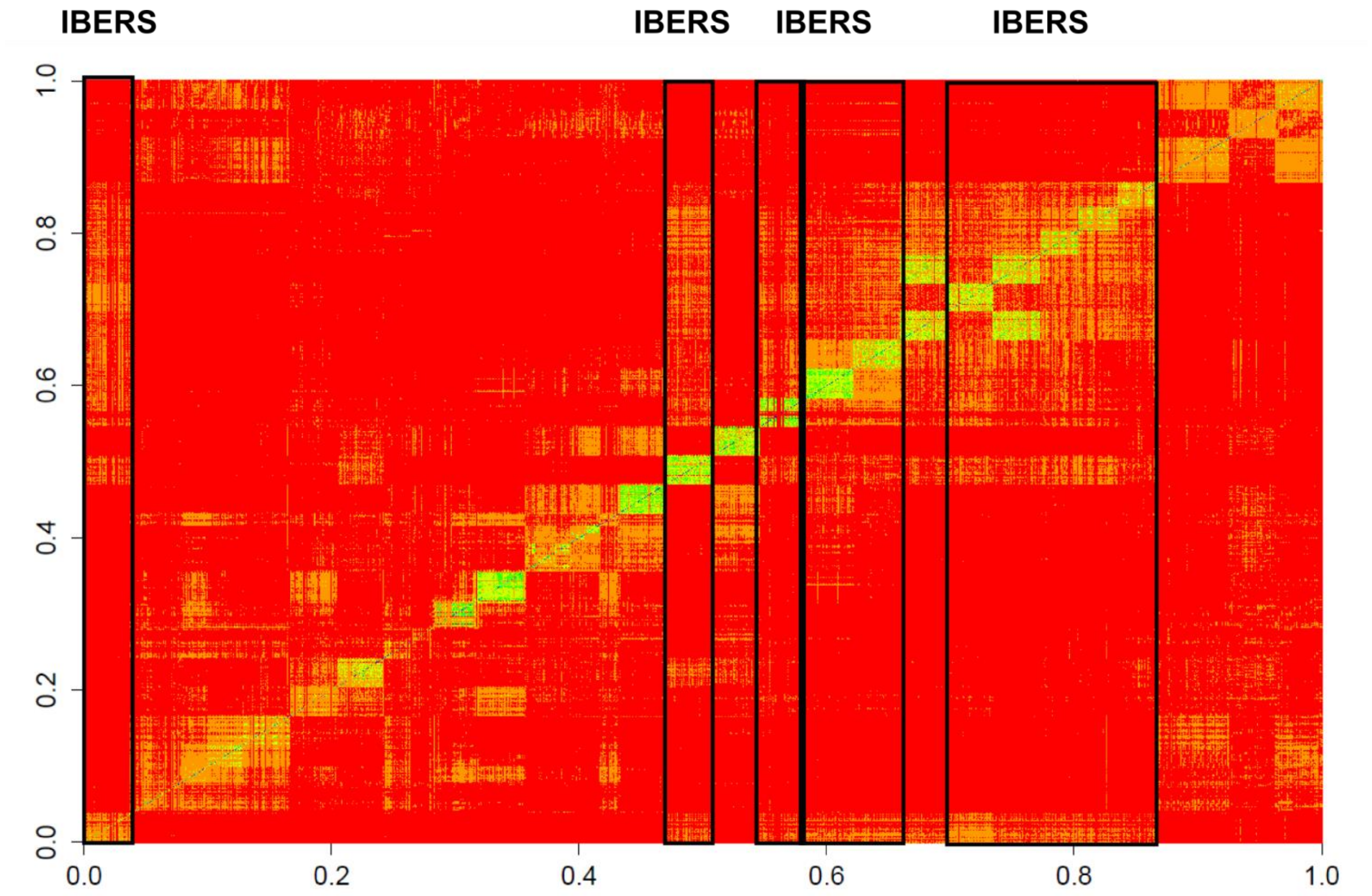

Supplementary Figure 3: Genomic relationship matrix. Genomic relationship matrix generated with SNP data, showing strong relationships between individual plants from the same family (individual plants are ordered according to the family they belong to, G01-G30). Plants originating from IBERS cultivars are highlighted.

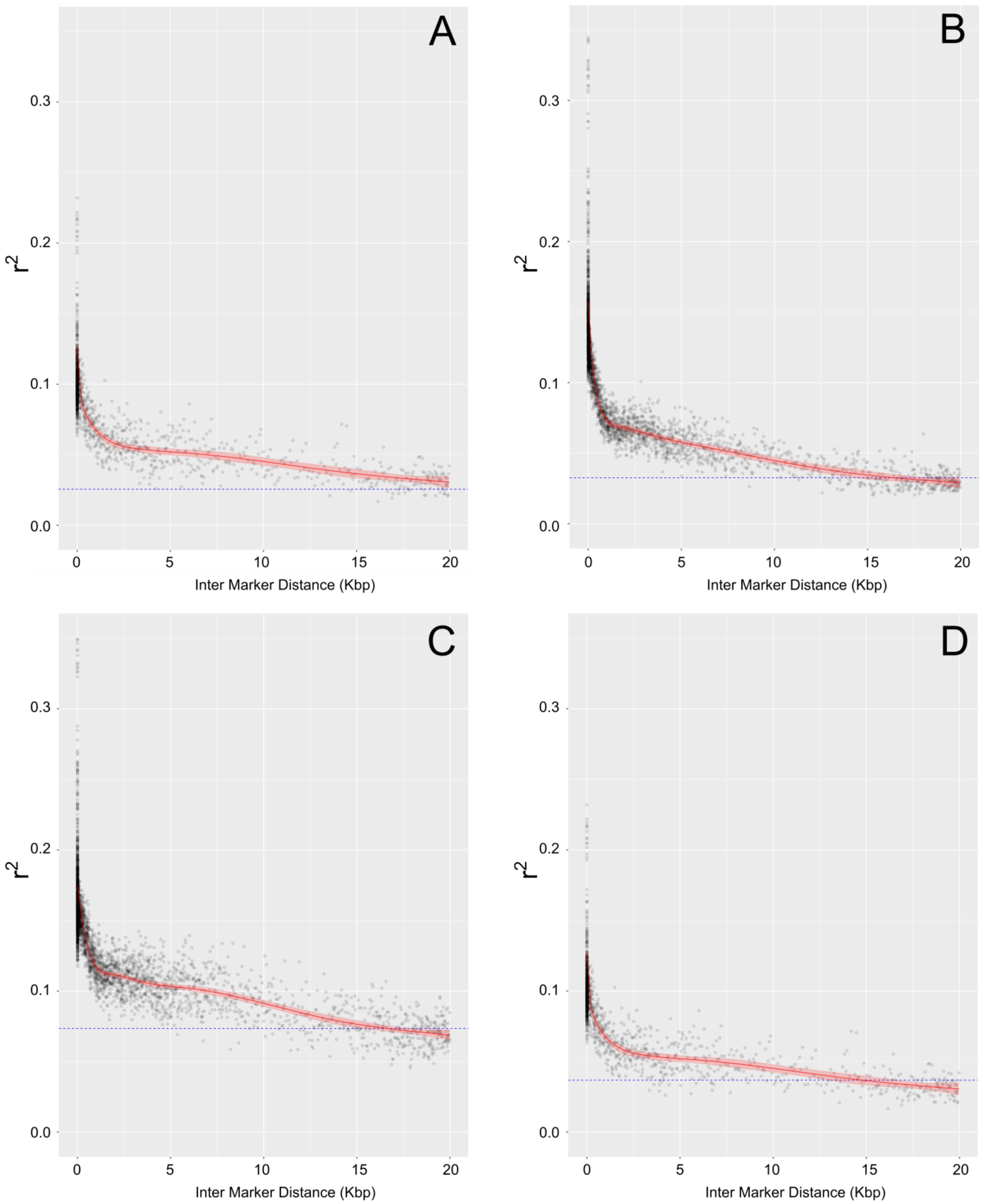

Supplementary Figure 4: Linkage Disequilibrium (LD) decay plots for complete population (A), full-sibs (B), half-sibs (C), and cultivars (D). LD was estimated between SNPs located within a single genomic scaffold and is the squared correlation of the allele counts (r^2). Estimates were ordered according to inter-marker distance. Mean r^2 and mean inter-marker distances were calculated every 1000 estimates and plotted. The blue line shows the mean r^2 calculated from 100,000 estimates between SNPs located on different genomic scaffolds.
